# Muscular resistance, hypertrophy and strength training equally reduce adiposity, inflammation and insulin resistance in mice with diet-induced obesity

**DOI:** 10.31744/einstein_journal/2020AO4784

**Published:** 2019-09-16

**Authors:** Janesca Mansur Guedes, Bruno Luiz da Silva Pieri, Thaís Fernandes Luciano, Schérolin de Oliveira Marques, Luiz Guilherme Antonacci Guglielmo, Claudio Teodoro de Souza

**Affiliations:** 1 Universidade Regional Integrada do Alto Uruguai e das Missões, Erechim, RS, Brazil.; 2 Universidade do Extremo Sul Catarinense, Criciúma, SC, Brazil.; 3 Universidade Federal de Santa Catarina, Florianópolis, SC, Brazil.; 4 Universidade Federal de Juiz de Fora, Juiz de Fora, MG, Brazil.

**Keywords:** Obesity, Exercise, Resistance training, Weight loss, Inflammation, Insulin/metabolism, Insulin resistance, Mice

## Abstract

**Objective:**

To evaluate the effect of three types of muscular resistance training on adiposity, inflammation levels and insulin activity in Swiss mice with fat-rich diet-induced obesity.

**Methods:**

Lean and obese male Swiss mice were selected and allocated to one of eight groups comprising eight mice each, as follows: standard diet + no training; standard diet + muscular resistance training; standard diet + hypertrophy training; standard diet + strength training; high-fat diet + no training; high-fat diet + muscular resistance training; high-fat diet + hypertrophy training; high-fat diet + strength training. The training protocol consisted of stair climbing for a 10-week period. Blood samples were collected for lactate analysis, glucose level measurement and insulin tolerance test. After euthanasia, adipose tissues were removed and weighed for adiposity index determination. Fragments of epididymal adipose tissue were then embedded for histological analysis or homogenized for tumor necrosis factor alpha level determination using the ELISA method.

**Results:**

Ausency of differences in total training volume and blood lactate levels overall emphasize the similarity between the different resistance training protocols. Body weight loss, reduced adipocyte area and lower adiposity index were observed in trained obese mice, regardless of training modality. Different training protocols also improved insulin sensitivity and reduced inflammation levels.

**Conclusion:**

Resistance training protocols were equally effective in reducing body fat, inflammation levels and insulin resistance in obese mice.

## INTRODUCTION

Obesity is a matter of growing concern given the rising prevalence of the condition and associations with several comorbidities, including *diabetes mellitus* .^( 1 )^ Obesity is a multifactorial condition involving inter-related biological, psychological, nutritional, hormone, economic, social, behavioral and environmental factors leading to body fat accumulation.^( 2 )^ Excessive caloric intake combined with low levels of physical activity stands out among factors contributing to excess body weight; therefore, lifestyle is a major, if not determining factor in prevention and ancillary treatment of these metabolic diseases.^( 3 )^

Obesity-related complications are partly associated with fat tissue changes. Fast developing fat tissue hypertrophy resulting from excessive triglyceride accumulation in response to nutrient overload and low levels of physical activity leads to fat tissue remodeling. Adipocyte hypertrophy is followed by macrophage infiltration and increasing levels of inflammation, with excessive release of pro-inflammatory cytokines, such as tumor necrosis factor alpha (TNF-α), interleukin 1 beta (IL-1β) and IL-6,^( 4 , 5 )^ and increased release of free fatty acids.^( 4 )^ Combined, these events lead to low-grade or subclinical inflammation.^( 6 , 7 )^ Saturated fatty acids (acting via toll−like receptors, TLR) and cytokines (acting via their respective receptors) may activate intracellular inflammatory signaling pathways involved in activation of proteins associated with inflammatory responses, such as c-Jun N-terminal kinases (JNK) and I Kappa B kinase (IKK), which may interfere with insulin activity.^( 8 )^ Activation of these serine kinases (JNK and IKK) may play a role in insulin receptor substrate function; once phosphorylated on serine, these substrates can no longer be phosphorylated on tyrosine by insulin receptors, contributing to resistance to insulin signal transduction via this pathway.^( 9 )^

Physical exercise is widely recommended by researchers for recovery and maintenance of physical and emotional health and well-being, leading to improved quality of life, gradual body weight loss, decreased levels of pro-inflammatory mediators and reversion of the insulin resistance, particularly in obese and type 2 diabetes (DM2) patients.^( 10 )^

Studies investigating the effects of aerobic training on modulation of pro-inflammatory molecules, such as JNK, TNF-α, IKK and others, and on improvement of insulin sensitivity have been published.^( 11 - 17 )^ Investigations in rats with fat-rich diet-induced obesity revealed that aerobic exercise reduces molecules in the pro-inflammatory pathway, and increases intracellular insulin signaling, leading to increased sensitivity to this hormone.^( 13 , 14 , 16 )^ However, further studies investigating the effects of different types of physical exercise on inflammatory markers associated with insulin resistance are warranted. Negative energy balance associated with progressive muscular resistance training and resulting changes in body fat distribution and muscle mass gain^( 18 )^ suggest this type of training may also have beneficial effects on adipokine levels and insulin sensitivity.

## OBJECTIVE

To analyze the effects of three types of muscular resistance training on adiposity, low-grade chronic inflammation and insulin sensitivity in mice with fat-rich diet-induced obesity.

## METHODS

### Animals

This project was conducted following approval by *Comissão de Ética em Uso de Animais* (CEUA) [Ethics Committee on Use of animals] of *Universidade do Extremo Sul Catarinense* (UNESC), protocol 053-2014-1. Male Swiss mice aged 4 weeks and weighing 25 g were obtained from the UNESC vivarium. Following environmental adaptation, mice were fed a standard or fat-rich diet. Mice were kept in collective cages under a 12-hour light-dark cycle; feed and water were provided *ad libitum* . The room temperature was kept at 20±2ºC. Mice were fed the fat-rich diet (PragSoluções Biociências, Jaú, SP, Brazil) for 17 weeks. Obesity and insulin resistance were confirmed based on body weight and insulin tolerance test (ITT) responses, respectively; mice were then allocated to one of eight experimental groups comprising eight mice each, as follows: standard diet + no training (DPNT); standard diet + muscular resistance training (DPTR); standard diet + hypertrophy training (DPTH); standard diet + strength training (DPTF); fat-rich diet + no training (DHNT); fat-rich diet + muscular resistance training (DHTR); fat-rich diet + hypertrophy training (DHTH); fat-rich diet + strength training (DHTF).

### Training protocols

Muscular resistance training protocols adopted in this study were according to Luciano et al.^( 19 )^ Mice with weights attached to their tails were submitted to stair climbing exercise consisting of repeated climbing of a vertical staircase to reach a 20×20×20cm compartment located at the top. Mice were taken back down with researcher’s assistance.

### Muscular resistance training protocol

Mice were submitted to muscular resistance training as follows: initial load corresponding to 10% of body mass (weeks 1 to 4) followed by progressive increase to 20% (weeks 4 to 6), 30% (weeks 6 to 8), then to 50% (weeks 8 to 10); 15 repetitions at 2-minute intervals, 5 days per week, for 10 weeks.

### Hypertrophy training protocol

Mice were submitted to hypertrophy training as follows: initial load corresponding to 25% of body mass (weeks 1 to 4) followed by progressive increase to 50% (weeks 4 to 7) then 75% (weeks 7 to 10); eight repetitions at 2-minute intervals, 5 days per week, for 10 weeks.

### Strength training protocol

Mice were submitted to strength training as follows: initial load corresponding to 50% of body mass (weeks 1 to 2) followed by progressive increase to 75% (weeks 2 to 3), 100% (weeks 3 to 5), 125% (weeks 5 to 7), 150% (weeks 7 to 9), then 175% (weeks 9 to 10); three to four repetitions at 2-minute intervals, 5 days per week, for 10 weeks.

### Training volume calculation

Training volume was compared among protocols as follows: repetitions *versus* series *versus* load^( 20 )^ expressed in joules.

### Lactate analysis

Blood samples for lactate analysis were collected in weeks one, four, seven and ten (samples 1, 2, 3 and 4, respectively), always at time point zero (zero minutes) after completion of the training session. A small incision was made at the tip of the tail; 15μL blood samples were then collected into microtubes containing 30μL of 1% sodium fluoride (NaF) and immediately frozen for future analysis. Lactate analysis was performed via the electroenzymatic method using a biochemical analyzer (Yellow Springs 2700S).

### Body weight and adiposity index

Body weight was recorded prior to start of training (week zero), then on weeks 5 and 10 of the training period. Mice were beheaded 48 hours after the last training session. Mesenteric, epididymal, retroperitoneal and perirenal fat tissues were dissected away and weighed for adiposity index determination (g/100g of body weight).

### Insulin tolerance test

Tests were performed after a 6-hour fasting period. The first blood sample was defined as test time point zero. Insulin (1U/kg of body weight) was then injected via the intraperitoneal route and blood samples collected from the tip of the tail at time points 5, 10, 15, 20, 25 and 30 minutes for blood glucose determination using a glucometer. Rate constant for glucose disappearance (k_ITT_) was calculated using the formula 0.693/t1/2. Glucose t1/2 was calculated from the slope of the least square analysis of serum glucose concentrations during the linear decay phase.

### Enzymatic immunosorbent assay (ELISA)

TNF-α was measured using ELISA; tests were run according manufacturer’s instructions (R&D Systems).

### Histologic analysis

Fragments of epididymal adipose tissue were collected and processed. Paraffin-embedded tissue samples were then cut into 5μm slices (microtome Hacker Edge SL-200). Slides were stained with hematoxylin and eosin (HE) for tissue architecture evaluation. Images were acquired using optical microscope (Nikon Eclipse Ti-U).

### Statistical analysis

Data were expressed as mean and standard error of the mean (SEM). Data normality and homogeneity of intergroup variance were investigated using the Shapiro-Wilk and the Levene test, respectively. The Student’s *t* test was used for comparisons limited to two groups (standard diet *versus* fat-rich diet). Comparisons involving all eight groups were based on one-way analysis of variance (ANOVA) followed by the Newman-Keuls post-hoc test. The level of significance was set at 5%; p<0.05. Statistical tests were performed using software (GraphPad Prism, version 7.0).

## RESULTS

### Effects of fat-rich diet on body weight and insulin sensitivity

Mice fed the fat-rich diet were heavier and had higher fasting insulin levels, lower k_ITT_ index and lower total glucose uptake (area under the curve) during the insulin tolerance test, suggesting lower insulin sensitivity ( Table 1 ).

**Table 1:** Body weight, glycemia, rate constant for glucose disappearance (kITT) and area under the glucose curve during insulin tolerance test, in Swiss mice fed a standard or fat-rich diet, prior training protocols (model characterization)

Parameter	Standard diet	Fat-rich diet
Body weight (g)	44.5±0.36	55.3±1.92^*^
Glycemia (mg/dL)	144.8±11.76	218.0±13.98^*^
k_ITT_ (%/minute)	4.60±0.53	2.12±0.53^*^
Area under the curve (mg/dL/minute)	2794±198.2	6650±399.3^*^

Results expressed as mean±standard error of the mean. *p<0.05 compared to groups fed the standard diet (Student’s *t* test).

### Resistance training volume and metabolic responses

**Figure 1:**
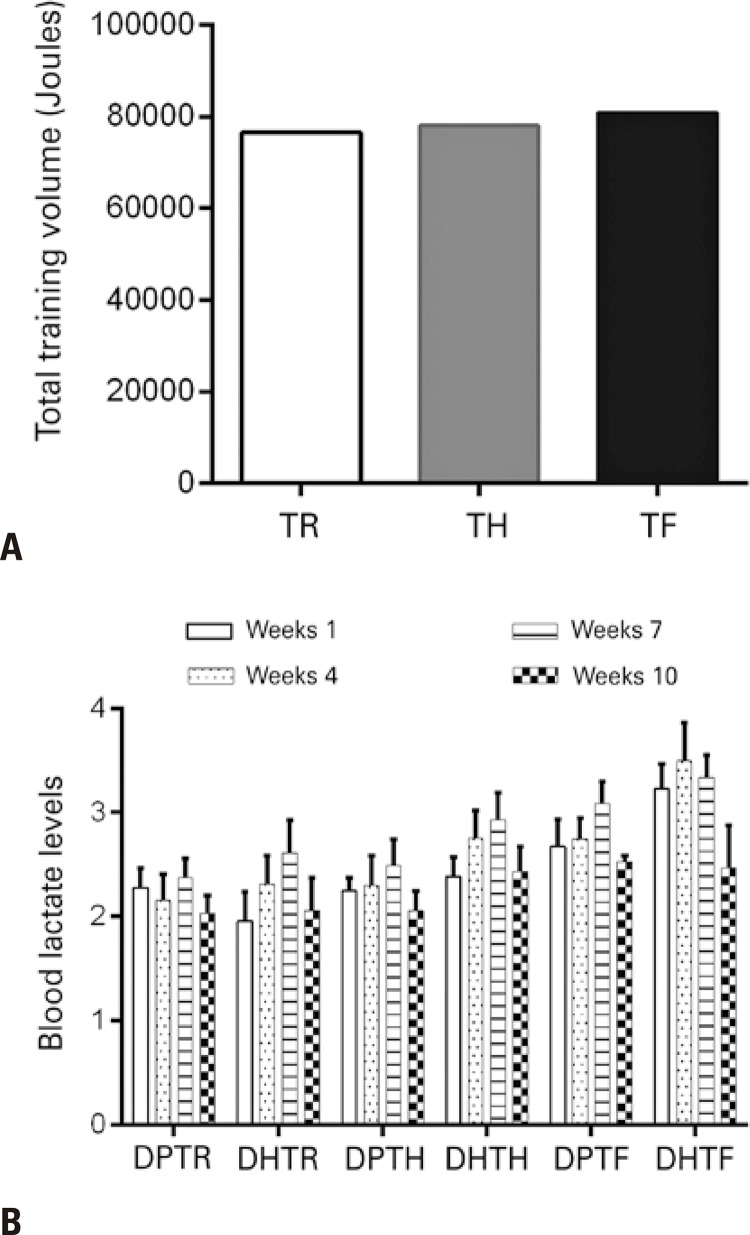
Total training volume and blood lactate levels. A) Total training volume. B) Intragroup and intergroup comparisons of blood lactate levels

TR: muscular resistance training; TH: hypertrophy training; TF: strength training; DPTR: standard diet + muscular resistance training; DHTR: fat-rich diet + muscular resistance training; DPTH: standard diet + hypertrophy training; DHTH: fat-rich diet + hypertrophy training; DPTF: standard diet + strength training; DHTF: fat-rich diet + strength training.

Total work volume and blood lactate levels were analyzed to investigate similarities between training protocols. Total training volume did not differ between training protocols ( Figure 1A ). Blood lactate levels were evaluated and no significant differences were found within (between samples) or between groups, emphasizing the similarity between training protocols ( Figure 1B ).

### Effects of resistance training on adiposity

**Figure 2:**
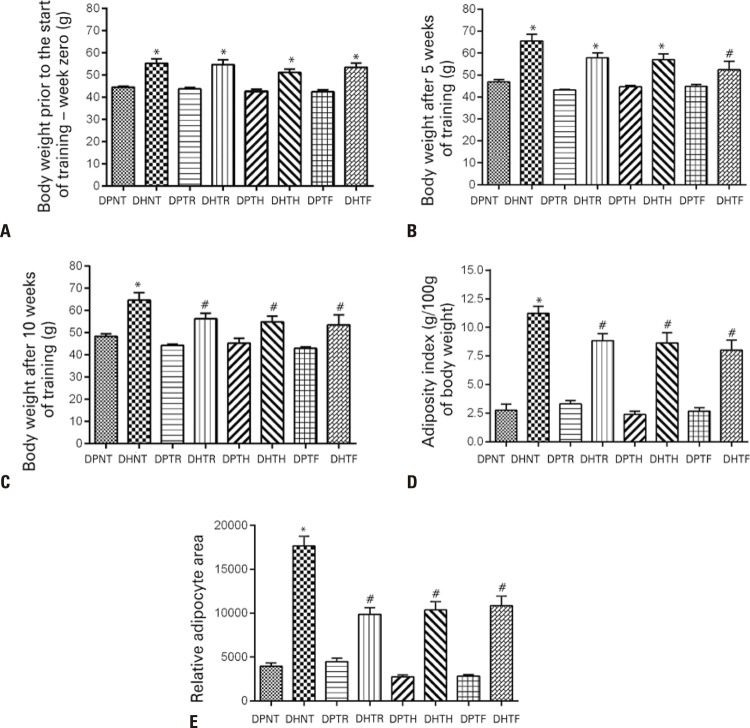
Body weight, adiposity index and adipocyte area of Swiss mice fed a standard or fat-rich diet and submitted to different muscular resistance training protocols. A) Body weight in week zero (1 day prior to the start of the training period). B) Body weight in week 5 (5 weeks after the start of the training period). C) Body weight in week 10 (1 day prior to euthanasia). D) Adiposity index. E) Relative adipocyte area

Mean initial body weight was similar within groups ( Figure 2A ). By week 5 of the training period, significant differences were noted between the DHTF and the DHNT Group only ( Figure 2B ). By week 10, mice submitted to either training protocol had lost weight compared to mice in the DHNT Group ( Figure 2C ). No significant differences were noted between trained mice fed the standard diet at any time point over the course of the training period, or between these and nontrained mice fed the same diet ( Figure 2C ).

* p<0.05 compared to groups fed the standard diet; ^#^ p<0.05 compared to the DHNT Group.

DPNT: standard diet + no training; DHNT: fat-rich diet + no training; DPTR: standard diet + muscular resistance training; DHTR: fat-rich diet + muscular resistance training; DPTH: standard diet + hypertrophy training; DHTH: fat-rich diet + hypertrophy training; DPTF: standard diet + strength training; DHTF: fat-rich diet + strength training.

According to the adiposity index, the fat-rich diet was able to effectively induce adiposity in the DHNT Group ( Figure 2D ). In contrast, training effectively reduced adiposity in mice fed the DH, regardless of training protocol. Adiposity did not differ between muscular resistance, hypertrophy and strength training groups; however, the adiposity index was lower in these compared to the DHNT Group ( Figure 2D ).

Aside from greater adiposity, mice fed the fat-rich diet had larger adipocyte area compared to mice in the DPNT Group ( Figure 2E ). The three types of resistance training equally reduced the adipocyte area compared to the DHNT Group ( Figure 2E ). Training did not change the adipocyte area in mice fed the standard diet.

### Effects of resistance training on glycemia and insulin sensitivity

**Figure 3:**
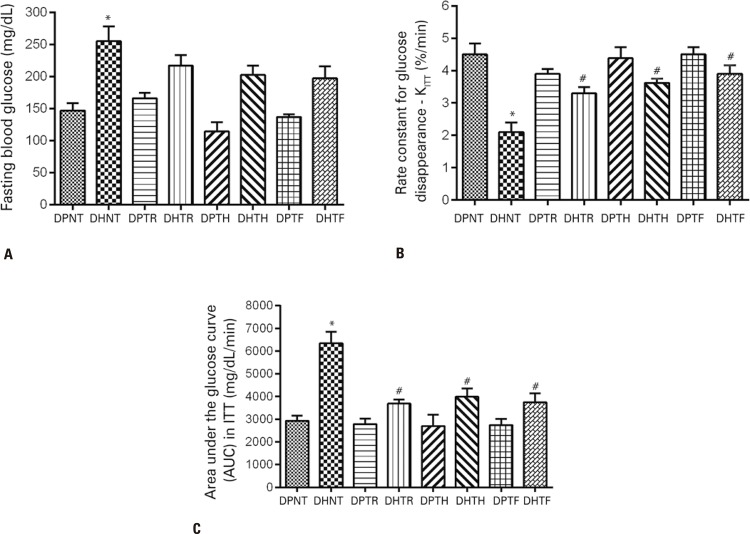
Glycemia and insulin sensitivity in Swiss mice fed a standard or fat-rich diet and submitted to different muscular resistance training protocols. A) Basal fasting blood glucose. B) Rate constant for glucose disappearance during the insulin tolerance test. C) Area under the glucose curve (AUC) during the insulin tolerance test

Basal fasting blood glucose levels did not differ following training ( Figure 3A ); however, mice fed the fat-rich diet had lower glucose decay rates ( Figure 3B ) and larger area under the curve ( Figure 3C ) compared to the mice in the DPNT Group. Muscular resistance, hypertrophy and strength training were equally associated with increased rate constant for glucose disappearance ( Figure 3B ) and smaller area under the curve ( Figure 3C ).

* p<0.05 compared to groups fed the standard diet; ^#^ p<0.05 compared to the DHNT Group.

DPNT: standard diet + no training; DHNT: fat-rich diet + no training; DPTR: standard diet + muscular resistance training; DHTR: fat-rich diet + muscular resistance training; DPTH: standard diet + hypertrophy training; DHTH: fat-rich diet + hypertrophy training; DPTF: standard diet + strength training; DHTF: fat-rich diet + strength training.

### Effects of muscular resistance training on TNF-α levels

**Figure 4:**
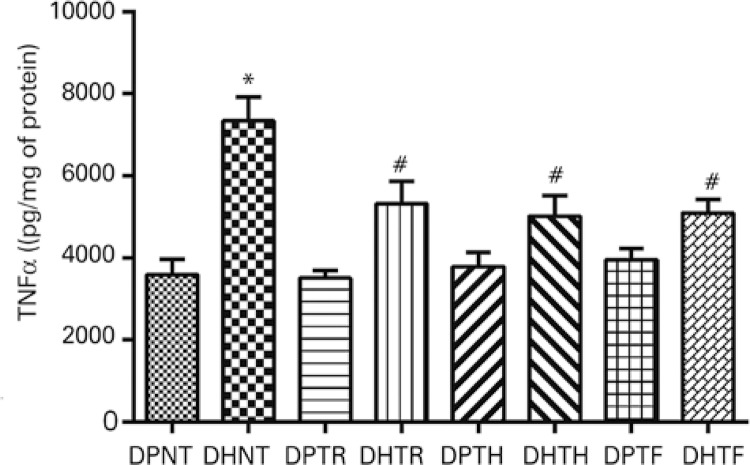
Tumor necrosis factor alpha (TNF-α) levels in the adipose tissue of Swiss mice fed a standard or fat-rich diet and submitted to different muscular resistance training protocols

TNF-α levels were evaluated in adipose tissue homogenate as a marker of low-grade chronic inflammation. Results revealed significant differences in TNF-α levels between nontrained mice fed the standard or the fat-rich diet ( Figure 4 ). In contrast, values were lower in trained groups fed the fat-rich diet compared to mice in the DHNT Group, regardless of training protocol ( Figure 4 ).

* p<0.05 compared to groups fed the standard diet; ^#^ p<0.05 compared to the DHNT Group.

DPNT: standard diet + no training; DHNT: fat-rich diet + no training; DPTR: standard diet + muscular resistance training; DHTR: fat-rich diet + muscular resistance training; DPTH: standard diet + hypertrophy training; DHTH: fat-rich diet + hypertrophy training; DPTF: standard diet + strength training; DHTF: fat-rich diet + strength training.

## DISCUSSION

Growing prevalence of obesity globally is a matter of concern demanding inter/multidisciplinary strategies. Some strategies include behavioral and nutritional changes, with emphasis on cardiopulmonary endurance exercise. However, the effects of muscular resistance exercise on health-related parameters is debatable.

Three different muscular resistance exercise protocols were evaluated in this study, all of which were equally effective in reducing body weight, adiposity index, adipocyte area, low-grade chronic inflammation and insulin resistance.

In this study, obese mice submitted to one of three types of training equally lost body weight and had lower adiposity index and adipocyte area. Similar findings have been reported in elsewhere. In a study by Leite et al., male Wistar rats with fat-rich diet-induced obesity submitted to a similar resistance training protocol (12 weeks of exercise consisting of climbing up a 1.1m staircase with weights attached to the tail) had significant body mass, lean mass and fat percentage reduction.^( 21 )^ Souza et al., reported reduced body mass, fat mass and fat percentage in Wistar rats submitted to muscular resistance training (stair climbing with weights attached to the tail, 8 to 12 times per session, 3 times per week, for 12 weeks) compared to sedentary obese rats.^( 22 )^ Speretta et al., compared the effects of muscular resistance (stair climbing) and cardiopulmonary endurance training (60 minutes of daily swimming with a load corresponding to 5% of body weight attached to the tail, 5 days per week, for 8 weeks) on adipocyte area.^( 23 )^ Muscular resistance training led to reduced adipocyte area in trained compared to sedentary obese mice; however, no differences were found between obese mice submitted to muscular resistance training or swimming).^( 23 )^ Mardare et al., also compared isometric strength training (clinging to a horizontal line with hands and feet for 3 minutes, three series at 1-minute intervals, five times per week) and cardiorespiratory endurance training (30 minutes of daily treadmill running at 80% of maximum oxygen consumption, 5 days per week, for 10 weeks) in C57BL/6 mice fed a fat-rich diet.^( 24 )^ According to authors of that study, both exercise protocols were equally able to reduced body weight compared to nontrained mice fed a standard diet.^( 24 )^

Similar adiposity reduction in mice submitted to different types of training suggests exercise protocols induced similar metabolic adaptations. It could be argued that changes in adiposity occurred in response to the effects of training on mice feeding behavior. Mice in this study were fed *ad libitum* ; however, other studies using the same training model failed to reveal significant food intake differences.^( 25 , 26 )^ Potential mechanisms underlying body mass and adiposity index reduction associated with strength and hypertrophy training (muscular resistance training) have been suggested, particularly due to muscle mass gain leading to higher basal metabolic rate and finally to body fat loss.^( 27 )^Further studies are warranted to investigate this topic.

Increased adiposity is a determining factor in insulin resistance development.^( 28 )^ Findings of this study revealed lower glucose decay rate and smaller area under the glucose curve in nontrained obese mice. Physical exercise significantly increased both parameters. Misra et al., submitted 30 obese patients to progressive muscular resistance training involving 6 muscle groups for a 3-month period, as follows: arm and shoulder flexion, finger extension and flexion, hip flexion, knee extension and heel elevation (two series of 10 repetitions each).^( 29 )^ The authors observed increased insulin sensitivity and reduced glycated hemoglobin levels in association with reduced adiposity.^( 29 )^ However, acute effects of muscular resistance exercise ( *i.e* ., regardless of adiposity reduction) have been reported. Acutely performed muscular resistance exercise (60% of 1 repetition maximum) induced significant insulin sensitivity improvement.^( 30 )^

Obesity induced by fat-rich diets promotes increased levels of inflammatory cytokines such as TNF-α. Therefore, increased levels of this cytokine were to be expected in this study. Mice fed the fat-rich diet actually had higher levels of TNF-α. On the other hand, it was hypothesized that muscular resistance training might impact TNF-α levels; in fact, physical exercise protocols in this study equally reduced levels of this cytokine. Effects of muscular resistance training consisting of stair climbing on TNF-α levels have been investigated.^( 23 )^ Speretta et al.,^( 23 )^ reported significant reduction in TNF-α levels in a study evaluating gene expression on the fat tissue of rats fed a standard or fat-rich diet and submitted to muscular resistance training.

This study has some limitations that will certainly be explored in future investigations, such as inclusion of pair-fed groups, assessment of basal metabolic rate and muscle fiber cross-sectional area, and investigation of molecules involved in insulin ( *e.g* ., phosphorylation of insulin receptor/IR and B kinase protein/Akt) and inflammatory ( *e.g* ., nuclear factor kappa B/NFκB and toll-like receptor/TLR) signal transduction, as well as other cytokines, including anti-inflammatory cytokines.

## CONCLUSION

Muscular resistance training protocols in this study were equally able to reduce body weight, adiposity index, adipocyte area and low-grade chronic inflammation, and to improve insulin resistance. Further investigations are warranted for increased understanding of the effects of muscular resistance exercise on body weight control. Still, this study provided significant contributions to the topic and introduced an alternative strategy for improved adherence of obese patients to exercise, since patients in this group may prefer muscular resistance to cardiorespiratory endurance exercise.

## References

[1] 1. NCD Risk Factor Collaboration (NCD-RisC). Trends in adult body-mass index in 200 countries from 1975 to 2014: a pooled analysis of 1698 population-based measurment studies with 19,2 milion participants. Lancet. 2016;387(10026):1377-96. Review. Erratum in: Lancet. 2016;387(10032):1998. 10.1016/S0140-6736(16)30054-X PMC7615134 27115820

[2] 2. Haslam DW, James WP. Obesity. Lancet. 2005;366(9492):1197-209. Review. 10.1016/S0140-6736(05)67483-1 16198769

[3] 3. Temelkova-Kurktschiev T, Stefanov T. Lifestyle and genetics in obesity and type 2 diabetes. Exp Clin Endocrinol Diabetes. 2012;120(1):1-6. Review. 10.1055/s-0031-1285832 21915815

[4] 4. Galic S, Oakhill JS, Steinberg GR. Adipose tissue as an endocrine organ. Mol Cell Endocrinol. 2010;316(2):129-39. Review. 10.1016/j.mce.2009.08.018 19723556

[5] 5. Alomar SY, Zaibi MS, Kępczyńska MA, Gentili A, Alkhuriji A, Mansour L, et al. PCR array and protein array studies demonstrate that IL-1β (interleukin-1β) stimulates the expression and secretion of multiple cytokines and chemokines in human adipocytes. Arch Physiol Biochem. 2015;121(5):187-93. 10.3109/13813455.2015.1087034 26471721

[6] 6. Gregor MG, Hotamisligil GS. Inflammatory mechanisms in obesity. Annu Rev Immunol. 2011;29:415-45. Review. 10.1146/annurev-immunol-031210-101322 21219177

[7] 7. Flehmig G, Scholz M, Klöting N, Fasshauer M, Tönjes A, Stumvoll M, et al. Identification of adipokine clusters related to parameters of fat mass, insulin sensitivity and inflammation. PLoS One. 2014;9(6):e99785. 10.1371/journal.pone.0099785 PMC4072672 24968098

[8] 8. Dandona P, Aljada A, Bandyopadhyay A. Inflammation: the link between insulin resistance, obesity and diabetes. Trends Immunol. 2004;25(1):4-7. Review. 10.1016/j.it.2003.10.013 14698276

[9] 9. Hotamisligil GS. Inflammation and metabolic disorders. Nature. 2006; 444(7121):860-7. Review. 10.1038/nature05485 17167474

[10] 10. Bays H, Blonde L, Rosenson R. Adiposopathy: how do diet, exercise and weight loss drug therapies improve metabolic disease in overweight patients? Expert Rev Cardiovasc Ther. 2006;4(6):871-95. Review. 10.1586/14779072.4.6.871 17173503

[11] 11. Ropelle ER, Pauli JR, editores. Paciente diabético: cuidados em educação física e esporte. Rio de Janeiro: MedBook; 2013.

[12] 12. Pauli JR, Cintra DE, Souza CT, Ropelle ER. [New mechanisms by which physical exercise improves insulin resistance in the skeletal muscle]. Arq Bras Endocrinol Metabol. 2009;53(4):399-408. Review. Portuguese. 10.1590/s0004-27302009000400003 19649376

[13] 13. Beavers KM, Brinkley TE, Nicklas BJ. Effect of exercise training on chronic inflammation. Clin Chim Acta. 2010;411(11-12):785-93. Review. 10.1016/j.cca.2010.02.069 PMC3629815 20188719

[14] 14. Da Silva AS, Pauli JR, Ropelle ER, Oliveira AG, Cintra DE, De Souza CT, et al. Exercise intensity, inflammatory signaling, and insulin resistance in obese rats. Med Sci Sports Exerc. 2010;42(12):2180-8. 10.1249/MSS.0b013e3181e45d08 20473230

[15] 15. Yaspelkis BB 3rd, Kvasha IA, Lessard SJ, Rivas DA, Hawley JA. Aerobic training reverses high-fat diet-induced pro-inflammatory signalling in rat skeletal muscle. Eur J App Physiol. 2010;110(4):779-88. 10.1007/s00421-010-1559-7 PMC5054743 20596724

[16] 16. Hussey SE, McGee SL, Garnham A, McConell GK, Hargreaves M. Exercise increases skeletal muscle GLUT4 gene expression in patients with type 2 diabetes. Diabetes Obes Metab. 2012;14(8):768-71. 10.1111/j.1463-1326.2012.01585.x 22340256

[17] 17. Marinho R, Moura LP, Rodrigues BA, Pauli LS, Silva AS, Ropelle EC, et al. Effects of different intensities of physical exercise on insulin sensitivity and protein kinase B/Akt activity in skeletal muscle of obese mice. einstein (São Paulo). 2014;12(1):82-9. 10.1590/S1679-45082014AO2881 PMC4898244 24728251

[18] 18. Lira FS, Lemos VA, Bittar IG, Caris AV, Dos Santos RV, Tufik S, et al. Physiological and cytokine response to acute exercise under hypoxic conditions: a pilot study. J Sports Med Phys Fitness. 2017;57(4):461-8. 10.23736/S0022-4707.16.06073-X 26796076

[19] 19. Luciano TF, Marques SO, Pieri BL, de Souza DR, Araújo LV, Nesi RT, et al. Responses of skeletal muscle hypertrophy in Wistar rats to different resistance exercise models. Physiol Res. 2017;66(2):317-23. 10.33549/physiolres.933256 27982685

[20] 20. Tan B. Manipulating resistance training program variables to optimize maximum strength in men: a review. Strength Cond Res.1999;13(3):289-304.

[21] 21. Leite RD, Durigan Rde C, de Souza Lino AD, de Souza Campos MV, Souza MD, Selistre-de-Araújo HS, et al. Resistence training may concomitantly benefit body composition, blood pressure and muscle MMP-2 activity on the left ventricle of high-fat fed diets rats. Metabolism. 2013;62(10):1477-84. 10.1016/j.metabol.2013.05.009 23790633

[22] 22. Souza MV, Leite RD, Lino AD, Marqueti RC, Bernardes CF, Araújo HS, et al. Resistance training improves body composition and increases matrix metalloproteinase 2 activity in biceps and gastrocnemius muscles of diet-induced obese rats. Clinics (Sao Paulo). 2014;69(4):265-70. 10.6061/clinics/2014(04)08 PMC3971365 24714835

[23] 23. Speretta GF, Rosante MC, Duarte FO, Leite RD, Lino AD, Andre RA, et al. The effects of exercise modalities on adiposity in obese rats. Clinics (Sao Paulo). 2012;67(12):1469-77. 10.6061/clinics/2012(12)19 PMC3521812 23295603

[24] 24. Mardare C, Krüger K, Liebisch G, Seimetz M, Couturier A, Ringseis R, et al. Endurance and Resistance Training Affect High Fat Diet-Induced Increase of Ceramides, Inflammasome Expression, and Systemic Inflammation in Mice. J Diabetes Res. 2016;2016:4536470. 10.1155/2016/4536470 PMC4691630 26788518

[25] 25. Jung S, Ahn N, Kim S, Byun J, Joo Y, Kim S, et al. The effect of ladder-climbing exercise on atrophy/hypertrophy-related myokine expression in middle-aged male Wistar rats. J Physiol Sci. 2015;65(6):515-21. 10.1007/s12576-015-0388-1 PMC10717129 26223833

[26] 26. Aguiar AF, Aguiar DH, Felisberto AD, Carani FR, Milanezi RC, Padovani CR, et al. Effects of creatine supplementation during resistance training on myosin heavy chain (MHC) expression in rat skeletal muscle fibers. J Strength Cond Res. 2010;24(1):88-96. 10.1519/JSC.0b013e3181aeb103 19816211

[27] 27. Schmitz KH, Jensen MD, Kugler KC, Jeffery RW, Leon AS. Strength training for obesity prevention in midlife women. Int J Obes Relat Metab Disord. 2003;27(3):326-33. 10.1038/sj.ijo.0802198 12629559

[28] 28. Tateya S, Kim F, Tamori Y. Recent advances in obesity-induced inflammation and insulin resistance. Front Endocrinol (Lausanne). 2013;4:93. 10.3389/fendo.2013.00093 PMC3737462 23964268

[29] 29. Misra A, Alappan NK, Vikram NK, Goel K, Gupta N, Mittal K, et al. Effect of supervised progressive resistance-exercise training protocol on insulin sensitivity, glycemia, lipids, and body composition in Asian Indians with type 2 diabetes. Diabetes Care. 2008;31(7):1282-7. 10.2337/dc07-2316 PMC2453659 18316394

[30] 30. Di Meo S, Iossa S, Venditti P. Improvement of obesity-linked skeletal muscle insulin resistance by strength and endurance training. J Endocrinol. 2017;234(3):R159-181. Review. 10.1530/JOE-17-0186 28778962

