# Supplementary material for: Muscular resistance, hypertrophy and strength training equally reduce adiposity, inflammation and insulin resistance in mice with diet-induced obesity
Source: Einstein (Sao Paulo). 2019 Sep 16;18:eAO4784. doi: 10.31744/einstein_journal/2020AO4784 (PMC6905165; doi:10.31744/einstein_journal/2020AO4784)
Supplement: Translation

# Translation: Exercícios físicos de resistência, hipertrofia e força muscular reduzem igualmente adiposidade, inflamação e resistência à insulina em camundongos obesos por dieta hiperlipídica

**Authors:** Janesca Mansur Guedes, Bruno Luiz da Silva Pieri, Thaís Fernandes Luciano, Schérolin de Oliveira Marques, Luiz Guilherme Antonacci Guglielmo, Claudio Teodoro de Souza

**Affiliations:** 1 Universidade Regional Integrada do Alto Uruguai e das Missões, Erechim, RS, Brasil.; 2 Universidade do Extremo Sul Catarinense, Criciúma, SC, Brasil.; 3 Universidade Federal de Santa Catarina, Florianópolis, SC, Brasil.; 4 Universidade Federal de Juiz de Fora, Juiz de Fora, MG, Brasil.

**Keywords:** Obesidade, Exercício, Treinamento de resistência, Perda de peso, Inflamação, Insulina/metabolismo, Resistência à insulina, Camundongos

## Abstract

**Objetivo:**

Avaliar os efeitos de três tipos de treinamentos de resistência na adiposidade, na inflamação e na ação da insulina em camundongos Swiss obesos por dieta hiperlipídica.

**Métodos:**

Camundongos Swiss machos magros e obesos foram selecionados e posteriormente separados em oito grupos com oito animais em cada: dieta padrão + não treinado; dieta padrão + treinamento de resistência muscular; dieta padrão + treinamento de hipertrofia; dieta padrão + treinamento de força; dieta hiperlipídica + não treinado; dieta hiperlipídica + treinamento de resistência muscular; dieta hiperlipídica + treinamento de hipertrofia; e dieta hiperlipídica + treinamento de força. O protocolo de treinamento consistiu em escaladas, por um período de 10 semanas. Amostras de sangue foram coletadas para análises de lactato, glicemia e teste de tolerância à insulina. Após eutanásia, os tecidos adiposos foram retirados e pesados para determinar o índice de adiposidade. Em seguida, parte do tecido adiposo epididimal foi emblocado para análises histológicas, e outra parte foi homogeneizada para análises de fator de necrose tumoral alfa por ELISA.

**Resultados:**

O volume total de treinamento e a concentração sanguínea de lactato não diferiram entre os três treinos resistidos, sugerindo similaridade entre eles. Nos animais obesos, as três modalidades de treinamento reduziram o peso corporal, a área adipocitária e o índice de adiposidade. Os três tipos de treinamentos ainda melhoraram a tolerância à insulina e reduziram a inflamação.

**Conclusão:**

Os protocolos de treinamento resistido foram igualmente efetivos em reduzir a adiposidade, a inflamação e a resistência à ação da insulina em camundongos obesos.

## INTRODUÇÃO

A obesidade tornou-se um tema de crescente preocupação, dado o importante aumento em sua prevalência e sua associação com inúmeras comorbidades, como o *diabetes mellitus* .^( 1 )^A etiologia da obesidade é multifatorial e envolve fatores biológicos, psicológicos, nutricionais, hormonais, econômicos, sociais, comportamentais e ambientais, os quais se inter-relacionam, resultando em acúmulo de gordura corporal.^( 2 )^Entre eles, o consumo excessivo de calorias associado ao baixo nível de atividade física é reconhecido como o que mais contribui para o excesso de peso corporal, sendo o estilo de vida crucial, senão determinante, para prevenção e adjuvante no tratamento dessas doenças metabólicas.^( 3 )^

As complicações decorrentes da obesidade estão associadas, em parte, com alterações no tecido adiposo. A rápida hipertrofia do tecido adiposo, decorrente do acúmulo excessivo de triglicerídeos em resposta à sobrecarga de nutrientes e a baixos níveis de atividade física, leva à sua remodelação. A hipertrofia dos adipócitos é seguida pela infiltração de macrófagos e pelo aumento da inflamação, com produção exacerbada de citocinas pró-inflamatórias, como fator de necrose tumoral alfa (TNF-α), interleucina 1 beta (IL-1β) e IL-6,^( 4 , 5 )^acompanhado pelo aumento da liberação de ácidos graxos livres.^( 4 )^Juntos, estes eventos levam ao desenvolvimento de um quadro de inflamação crônica de baixo grau ou subclínica.^( 6 , 7 )^Os ácidos graxos saturados, agindo por meio de receptor do tipo *toll* (TLR - *toll-like receptor* ) e as citocinas (agindo por meio de seus receptores) podem ativar vias de sinalização inflamatórias intracelulares, como as que acionam proteínas de respostas inflamatórias, como c-Jun quinase N-terminal (JNK) e I Kappa B quinase (IKK), que podem interferir na ação da insulina.^( 8 )^A ativação dessas serinas quinases (JNK e IKK) pode interferir na funcionalidade dos substratos do receptor de insulina, que, uma vez fosforilados em serina, têm comprometida a possibilidade de serem fosforilados em tirosina pelo receptor de insulina, contribuindo para a resistência à transdução do sinal da insulina, por meio dessa via.^( 9 )^

Exercícios físicos são amplamente recomendados por pesquisadores para recuperar e manter a saúde e o bem-estar físico e emocional, garantindo ao indivíduo melhora da qualidade de vida, com a perda gradativa de peso corporal, a diminuição de mediadores pró-inflamatórios e a reversão do quadro de resistência à ação da insulina, principalmente em pacientes obesos e diabéticos do tipo 2 (DM2).^( 10 )^

Neste sentido, estudos sobre os efeitos do treinamento físico aeróbio na modulação das moléculas pró-inflamatórias, como JNK, TNF-α e IKK, entre outras, e a melhora da sensibilidade a insulina têm sido publicados.^( 11 - 17 )^Pesquisas realizadas com ratos com obesidade induzida por dieta rica em gordura relatam que o exercício aeróbio reduz moléculas da via pró-inflamatória e aumenta a sinalização intracelular da insulina, elevando a sensibilidade a esse hormônio.^( 13 , 14 , 16 )^No entanto, mais estudos analisando os efeitos dos diferentes tipos de treinamento físico sobre marcadores inflamatórios associados à resistência à insulina são necessários. Como o treinamento resistido progressivo promove um balanço energético negativo, alterando a distribuição da gordura corporal e aumentando a massa muscular,^( 18 )^acredita-se que ele tenha efeito benéficos também sobre os níveis de adipocinas e na sensibilidade à insulina.

## OBJETIVO

Analisar os efeitos de três tipos de treinamento resistido sobre a adiposidade, a inflamação crônica de baixo grau e a sensibilidade à insulina em camundongos com obesidade induzida por dieta hiperlipídica.

## MÉTODOS

### Animais

Este projeto foi executado após aprovação da Comissão de Ética em Uso de Animais (CEUA) da Universidade do Extremo Sul Catarinense (UNESC), sob protocolo 053-2014-1. Os camundongos Swiss machos foram recebidos do biotério da UNESC com 4 semanas de idade e peso inicial de 25g. Após adaptação ao ambiente, foram alimentados com dieta padrão e dieta rica em gordura. Os animais foram mantidos em gaiolas coletivas, em um ciclo claro e escuro de 12 horas, e receberam comida e água *ad libitum* . O ambiente foi mantido à temperatura de 20±2ºC. Os camundongos foram alimentados por 17 semanas com dieta hiperlipídica (PragSoluções Biociências, Jaú, SP, Brasil). Após comprovadas a obesidade (verificada por peso corporal) e a resistência à insulina (por meio do teste de tolerância à insulina – ITT), os camundongos foram subdivididos em oito grupos experimentais, com oito animais por grupo: dieta padrão + não treinado (DPNT); dieta padrão + treinamento de resistência muscular (DPTR); dieta padrão + treinamento de hipertrofia (DPTH); dieta padrão + treinamento de força (DPTF); dieta hiperlipídica + não treinado (DHNT); dieta hiperlipídica + treinamento de resistência muscular (DHTR); dieta hiperlipídica + treinamento de hipertrofia (DHTH); e dieta hiperlipídica + treinamento de força (DHTF).

### Protocolos de treinamentos

Neste estudo, foi utilizado o protocolo de treinamento resistido adaptado de Luciano et al.^( 19 )^O protocolo consistiu nos animais escalarem uma escada vertical com pesos atados em suas caudas até alcançarem o topo da escada. No topo, os camundongos alcançavam um compartimento (20×20×20cm); com a ajuda do pesquisador, os camundongos eram novamente colocados na parte inferior da escada, e todo o processo se iniciava novamente.

### Protocolo de treinamento de resistência muscular

Os animais foram submetidos ao treinamento de resistência, no qual a carga inicial foi de 10% da massa corporal (primeira à quarta semana), aumentando progressivamente para 20% (quarta à sexta semana), 30% (sexta à oitava semana) e 50% (oitava à décima semana), com 15 repetições, 2 minutos de intervalo, 5 dias por semana, durante 10 semanas.

### Protocolo de treinamento de hipertrofia

Os animais foram submetidos ao treinamento de hipertrofia, no qual a carga inicial foi de 25% da massa corporal (primeira à quarta semana), aumentando progressivamente para 50% (quarta à sétima semana) e 75% (sétima à décima semana), com oito repetições, 2 minutos de intervalo, 5 dias por semana, durante 10 semanas.

### Protocolo de treinamento de força

Os animais foram submetidos ao treinamento de força, no qual a carga inicial foi de 50% da massa corporal (primeira à segunda semana), aumentando progressivamente para 75% (segunda à terceira semana), 100% (terceira à quinta semana), 125% (quinta à sétima semana), 150% (sétima à nona semana) e 175% (nona à décima semana), com três a quatro repetições, 2 minutos de intervalo, 5 dias por semana, durante 10 semanas.

### Cálculo do volume de treino

O volume de treinamento foi comparado utilizando-se o produto das repetições *versus* séries *versus* carga,^( 20 )^expresso em joules.

### Análise do lactato

A coleta de sangue para análise de lactato foi realizada em quatro momentos diferentes: na primeira (coleta 1), quarta (coleta 2), sétima (coleta 3) e décima (coleta 4) semanas. Foi realizada sempre no tempo zero minuto, após fim da sessão de treino. Após pequeno corte na extremidade da cauda do animal, amostras de 15μL de sangue foram coletadas e depositadas em microtubos contendo 30μL de fluoreto de sódio (NaF) a 1%, sendo imediatamente congeladas, para posterior análise das concentrações sanguíneas de lactato, pelo método eletroenzimático, em analisador bioquímico (Yellow Springs 2700S).

### Peso corporal e índice de adiposidade

O peso corporal foi registrado na semana zero (antes de começar o treinamento), na semana 5 e na semana 10 de treinamento. Após 48 horas da última sessão de treinamento, os animais foram mortos por decapitação, os tendo os tecidos adiposos mesentérico, epididimal, retroperitoneal e perirenal dissecados e pesados para determinação do índice de adiposidade (g/100g de peso corporal).

### Teste de tolerância à insulina

O teste foi realizado após 6 horas de jejum. A primeira coleta de sangue representou o tempo zero do teste. Em seguida, injetou-se insulina (1U/kg de peso corporal) intraperitonealmente, e amostras do sangue foram coletadas pela cauda nos tempos 5, 10, 15, 20, 25 e 30 minutos, para a determinação da glicose sanguínea por meio de glicosímetro. Calculou-se a velocidade constante do decaimento da glicose (k_ITT_) por meio da fórmula 0,693/t1/2. O t1/2 da glicose foi calculado a partir da curva da análise dos mínimos quadrados da concentração da glicose sérica, durante a fase de decaimento linear.

### Ensaio de imunoabsorbância enzimática (ELISA)

A dosagem de TNF-α foi realizada pelo método ELISA, segundo as especificações do fabricante (R&D Systems).

### Análise histológica

Fragmentos do tecido adiposo epididimal foram coletados, processados, incluídos em blocos de parafina e, posteriormente, seccionados em cortes de 5μm (micrótomo Hacker Edge SL-200). As lâminas foram coradas com coloração de hematoxilina e eosina (HE), a fim de avaliar a histoarquitetura do tecido. A aquisição das imagens foi realizada por microscópio óptico (Nikon Eclipse Ti-U).

### Análise estatística

Os dados foram expressos como média e erro padrão da média (EPM). As variáveis foram analisadas quanto à normalidade da distribuição usando o teste de Shapiro-Wilk, e a homogeneidade de variância foi avaliada entre os grupos por meio do teste de Levene. Quando comparados apenas dois grupos (dieta padrão *versus* dieta hiperlipídica), a ferramenta utilizada foi o teste *t de* Student. Quando comparados os oito grupos, a ferramenta utilizada foi a análise de variância (ANOVA) *one-way* , seguida pelo teste *post-hoc* de Newman-Keuls. O nível de significância estabelecido foi 5%; p<0,05. O programa GraphPad Prism, versão 7.0, foi utilizado para a aplicação dos testes estatísticos.

## RESULTADOS

### Efeitos da dieta hiperlipídica sobre o peso corporal e a sensibilidade à insulina

Os animais alimentados com dieta hiperlipídica apresentaram maiores pesos corporais e de glicemia de jejum, menor taxa de k_ITT_e menor quantidade total de absorção de glicose (medida pela área sob a curva) durante o teste de tolerância à insulina, indicando menor sensibilidade à insulina ( Tabela 1 ).

**Tabela 1:** Peso corporal, glicemia, constante de decaimento da glicose (kITT) e área sob a curva da glicose durante o teste de tolerância à insulina de camundongos Swiss alimentados com dieta padrão e com dieta hiperlipídica antes do treinamento físico resistido (caracterização do modelo)

Parâmetro	Dieta padrão	Dieta hiperlipídica
Peso corporal (g)	44,5±0,36	55,3±1,92^*^
Glicemia (mg/dL)	144,8±11,76	218,0±13,98^*^
k_ITT_(%/minuto)	4,60±0,53	2,12±0,53^*^
Área sob a curva (mg/dL/minuto)	2794±198,2	6650±399,3^*^

Resultados estão expressos como média±erro padrão da média. * p<0,05 em comparação aos grupos dieta padrão (Test *t* de Student).

### Volume e respostas metabólicas dos treinos resistidos

Objetivando saber as similaridades dos treinos, analisou-se o volume total de trabalho e os níveis sanguíneos de lactato. O volume total de treinamento não diferiu entre os três tipos de treinamento ( Figura 1A ). Os níveis de lactato sanguíneo foram avaliados. Nenhuma diferença estatística nos níveis de lactato sanguíneo foi observada nas comparações intragrupo (entre as coletas) e intergrupo, demonstrando similaridade entre os treinos ( Figura 1B ).

**Figura 1:** Volume total de treinamento e níveis de lactato sanguíneo. A) Volume total de treinamento. B) Comparação intragrupo e intergrupo dos níveis sanguíneo de lactato

TR: treinamento de resistência muscular; TH: treinamento de hipertrofia muscular; TF: treinamento de força muscular; DPTR: dieta padrão + treinamento de resistência muscular; DHTR: dieta hiperlipídica + treinamento de resistência muscular; DPTH: dieta padrão + treinamento de hipertrofia; DHTH: dieta hiperlipídica + treinamento de hipertrofia; DPTF: dieta padrão + treinamento de força; DHTF: dieta dieta hiperlipídica + treinamento de força.

### Efeitos do treinamento resistido sobre a adiposidade

O peso médio inicial dos animais demonstra pesos similares intragrupos ( Figura 2A ). Na semana 5 de treinamento, somente o Grupo DHTF apresentou diferença significativa quando comparado ao Grupo DHNT ( Figura 2B ). Na semana 10, os camundongos apresentaram reduzido peso corporal em todos os três tipos de treinamento, quando comparados com o Grupo DHNT ( Figura 2C ). Os camundongos alimentados com dieta padrão, em nenhum momento do período de treinamento, demonstraram diferença estatística entre os grupos ou comparado ao grupo não treinado ( Figura 2C ).

**Figura 2:** Peso corporal, índice de adiposidade e área de adipócito de camundongos Swiss alimentados com dieta padrão e com dieta hiperlipídica submetidos a diferentes protocolos de treinamento físico resistido. A) Peso corporal na semana zero (1 dia antes do início do período do treinamento). B) Peso corporal na semana 5 (5 semanas após início do período do treinamento). C) Peso corporal na semana 10 (1 dia antes da eutanásia). D) Índice de adiposidade. E) Área relativa dos adipócitos

* p<0,05 em comparação aos grupos dieta padrão;^#^p<0,05 em comparação ao Grupo DHNT.

DPNT: dieta padrão + não treinado; DHNT: dieta hiperlipídica + não treinado; DPTR: dieta padrão + treinamento de resistência muscular; DHTR: dieta hiperlipídica + treinamento de resistência muscular; DPTH: dieta padrão + treinamento de hipertrofia; DHTH: dieta hiperlipídica + treinamento de hipertrofia; DPTF: dieta padrão + treinamento de força; DHTF: dieta dieta hiperlipídica + treinamento de força.

O índice de adiposidade mostrou que a dieta hiperlipídica foi eficiente em induzir adiposidade no Grupo DHNT ( Figura 2D ). Por outro lado, os três tipos de treinamento foram eficazes em reduzir a adiposidade em camundongos alimentados com DH. A adiposidade não diferiu entre os grupos de treinamento de resistência muscular, hipertrofia e força, mas estes grupos apresentaram índice de adiposidade menor em comparação com o Grupo DHNT ( Figura 2D ).

Os animais do grupo alimentado com dieta hiperlipídica não só apresentaram maior adiposidade como também maior área adipocitária quando comparados ao Grupo DPNT ( Figura 2E ). Os três tipos de treinamento resistido reduziram de maneira igualitária a área adipocitária quando comparados ao Grupo DHNT ( Figura 2E ). Os treinamentos não alteraram a área adipocitária nos animais alimentados com dieta padrão.

### Efeitos do treinamento resistido sobre a glicemia e a sensibilidade à insulina

Após o treinamento, apesar de não ocorrer diferença nos valores de glicemia basal de jejum ( Figura 3A ), os animais alimentados com dieta hiperlipídica apresentaram menores valores da taxa de decaimento da glicose ( Figura 3B ) e maior área sob a curva ( Figura 3C ) quando comparados com o Grupo DPNT. Os treinamentos de resistência muscular, hipertrofia e de força aumentaram a constante de decaimento ( Figura 3B ) e reduziram a área sob a curva ( Figura 3C ) de maneira igualitária.

**Figura 3:** Glicemia e sensibilidade à insulina de camundongos Swiss alimentados com dieta padrão e com dieta hiperlipídica submetidos a diferentes protocolos de treinamento físico resistido. A) Glicose sanguínea basal de jejum. B) Constante de decaimento da glicose durante o teste de tolerância à insulina. C) Área sob a curva (ASC) da glicose durante o teste de tolerância à insulina

* p<0,05 em comparação aos grupos dieta padrão;^#^p<0,05 em comparação ao Grupo DHNT.

DPNT: dieta padrão + não treinado; DHNT: dieta hiperlipídica + não treinado; DPTR: dieta padrão + treinamento de resistência muscular; DHTR: dieta hiperlipídica + treinamento de resistência muscular; DPTH: dieta padrão + treinamento de hipertrofia; DHTH: dieta hiperlipídica + treinamento de hipertrofia; DPTF: dieta padrão + treinamento de força; DHTF: dieta dieta hiperlipídica + treinamento de força; k_ITT_: constante de decaimento da glicose.

### Efeitos do treinamento resistido sobre os níveis de TNF-α

Avaliaram-se os níveis de TNF-α em homogenato de tecido adiposo como marcador de inflamação crônica de baixo grau. Os resultados mostram diferença significativa entre os grupos dieta padrão e hiperlipídica não treinados em relação aos níveis de TNF-α ( Figura 4 ). Por outro lado, os valores foram igualmente reduzidos nos grupos dieta hiperlipídica que realizaram treinamentos quando comparados ao Grupo DHNT ( Figura 4 ).

**Figura 4:** Níveis de fator de necrose tumoral alfa (TNF-α) em tecido adiposo de camundongos Swiss alimentados com dieta padrão e com dieta hiperlipídica submetidos a diferentes protocolos de treinamento físico resistido

* p<0,05 em comparação aos grupos dieta padrão;^#^p<0,05 em comparação ao Grupo DHNT.

DPNT: dieta padrão + não treinado; DHNT: dieta hiperlipídica + não treinado; DPTR: dieta padrão + treinamento de resistência muscular; DHTR: dieta hiperlipídica + treinamento de resistência muscular; DPTH: dieta padrão + treinamento de hipertrofia; DHTH: dieta hiperlipídica + treinamento de hipertrofia; DPTF: dieta padrão + treinamento de força; DHTF: dieta dieta hiperlipídica + treinamento de força.

## DISCUSSÃO

A crescente prevalência global da obesidade é motivo de preocupação e exige estratégias inter/multidisciplinares. Algumas das estratégias incluem mudanças nos aspectos comportamentais e nutricionais, destacando o exercício físico de resistência cardiopulmonar. No entanto, muito ainda se discute em relação aos efeitos do exercício físico resistido sobre parâmetros relacionados à obesidade.

Neste estudo, avaliaram-se três diferentes protocolos de exercícios físicos resistido, e observou-se que estes foram igualmente efetivos em reduzir o peso corporal; o índice de adiposidade e área adipocitária; a inflamação crônica de baixo grau; e a resistência à insulina.

No presente estudo, os camundongos obesos submetidos aos três tipos de treinos reduziram igualmente o peso corporal, o índice de adiposidade e a área adipocitária. Resultados similares têm sido observados em estudos prévios. Utilizando treino resistido similar ao do presente estudo (12 semanas subindo escada de 1,1m com peso atado à cauda) em ratos Wistar machos obesos por dieta hiperlipídica, Leite et al., observaram que os animais apresentaram significativa redução de massa corporal, massa magra e percentual de gordura.^( 21 )^Utilizando treino resistido de subida em escada com peso atrelado a cauda, subindo de 8 a 12 vezes por sessão, com frequência de 3 sessões por semana, durante 12 semanas, Souza et al., registraram redução na massa corporal, massa de gordura e percentual de gordura quando comparado a ratos Wistar obeso sedentário.^( 22 )^Speretta et al., compararam os efeitos do treino resistido (subida em escada) com treino de resistência cardiopulmonar (natação, 60 minutos por dia, 5 dias por semana, durante 8 semanas, com sobrecarga de 5% do peso corporal atado à cauda) quanto à área adipocitária.^( 23 )^O treino resistido reduziu a área adipocitária quando comparado com o sedentário obeso, mas não houve diferença entre os diferentes tipos de treinamento (subida em escada versus natação).^( 23 )^Ainda, Mardare et al., compararam treino de força isométrica (agarrados com patas dianteira e traseiras em um fio horizontal por 3 minutos, três séries com intervalo de 1 minutos, cinco vezes por semana) e de resistência cardiorrespiratória (corrida a 80% do consumo máximo de oxigênio em esteira, 30 minutos por dia, 5 dias por semana, durante 10 semanas) em camundongos C57BL/6 tratados com dieta hiperlipídica.^( 24 )^Os autores observaram que os dois protocolos de exercício reduziram igualmente o peso corporal em relação ao grupo em dieta padrão não treinado.^( 24 )^

A redução da adiposidade ocorreu igualitariamente apesar das diferentes características de cada tipo de treinamento, sugerindo que os protocolos têm adaptações metabólicas similares. Seria possível pensar que as alterações na adiposidade decorreriam de efeitos dos treinos sobre o padrão alimentar dos animais. Apesar de, no presente estudo, a alimentação ter sido realizada de maneira livre ( *ad libitum* ), estudos utilizando o mesmo modelo de treino mostraram não ocorrer alteração significativa na ingestão alimentar.^( 25 , 26 )^Alguns sugerem potenciais mecanismos para a redução de massa corporal e índice de adiposidade em treinamentos de força e hipertrofia (treino resistido), principalmente por haver aumento da massa muscular, levando ao aumento na taxa metabólica basal e, assim, resultando na diminuição da gordura corporal.^( 27 )^Para verificar isso, estudos adicionais são necessários.

O aumento da adiposidade é um fator determinante para o desenvolvimento de resistência à insulina.^( 28 )^De fato, os resultados mostram redução no decaimento da glicose e na área sob a curva da glicose no grupo obeso não treinado. Os exercícios físicos significativamente aumentam ambos os parâmetros. Misra et al., submeterem 30 pacientes obesos, por 3 meses, ao treino resistido progressivo que envolvia 6 grupos musculares: flexão de braço e ombro, extensão e flexão dos dedos, flexão de quadril, extensão do joelho e elevação de calcanhar (duas séries de 10 repetições cada).^( 29 )^Os autores observaram aumento na sensibilidade à insulina e redução da hemoglobina glicada associados à reduzida adiposidade.^( 29 )^No entanto, os efeitos agudos do exercício resistido, ou seja, independente da redução da adiposidade, têm sido relatado. Exercício resistido realizado de maneira aguda (60% de 1 repetição máxima induziu significativa melhora na sensibilidade à insulina.^( 30 )^

O modelo de obesidade induzido por dieta rica em gordura constitui situação de aumento de citocinas inflamatórias, como o TNF-α. Assim, no presente estudo, esperava-se aumento dessa citocina. De fato, camundongos alimentados com dieta hiperlipídica apresentaram maiores níveis de TNF-α. Por outro lado, hipotetizou-se que os treinos resistidos poderiam ter tido algum efeito sobre os níveis de TNF-α. De fato, os três tipos de exercícios físicos igualmente reduziram os níveis dessa citociona. Os efeitos do treino resistido de subida em escada sobre níveis de TNF-α têm sido estudados.^( 23 )^Speretta et al.,^( 23 )^avaliaram a expressão gênica no tecido adiposo de ratos submetidos ao treinamento resistido alimentados com dieta padrão e hiperlipídica e observaram redução significativa nos níveis TNF-α.

O presente estudo possui algumas limitações que certamente serão exploradass em futuras investigações, tais como acrescentar grupos alimentados com ração pareada ( *pair feed* ); avaliar a taxa metabólica basal; avaliar a área transversa da fibra muscular; avaliar moléculas envolvidas na transdução do sinal insulínico, como fosforilação do receptor de insulina (IR) e a proteína quinase B (Akt) avaliar moléculas envolvidas na transdução do sinal infamatório, como fator nuclear kappa B (NFκB) e TLR e avaliar outras citocinas, incluindo as anti-inflamatórias.

## CONCLUSÃO

Os três protocolos de treinamento resistido reduziram igualmente o peso corporal, o índice de adiposidade, a área adipocitária, a inflamação crônica de baixo grau e a melhora do quadro de resistência à insulina. Mais investigações são necessárias para aumentar o conhecimento acerca dos efeitos do exercício resistido sobre controle ponderal. No entanto, o presente estudo mostrou considerável contribuição a esta questão e sugere opção para aderência, uma vez que pacientes obesos podem preferir praticar exercícios físicos resistidos a cardiorrespiratórios.
